# Micro Electrochemical Machining of Array Micro-Grooves Using In-Situ Disk Electrode Fabricated by Micro-WEDM

**DOI:** 10.3390/mi11010066

**Published:** 2020-01-07

**Authors:** Yukui Wang, Han Wang, Yuxin Zhang, Xiaolong He, Zhenlong Wang, Guanxin Chi, Xiang Chen, Mingshan Song

**Affiliations:** 1 School of Mechatronics Engineering, Harbin Institute of Technology, Harbin 150001, China; 2 Key Laboratory of Micro-Systems and Micro-Structures Manufacturing of Ministry of Education, Harbin Institute of Technology, Harbin 150001, China; 3 KEDE Numerical Control Co., Ltd., Dalian 116000, China

**Keywords:** disk electrode, micro-WEDM, in-situ, micro-ECM, array micro-grooves

## Abstract

This paper develops an array micro-grooves manufacturing method using micro electrochemical machining (ECM) with disk electrode, which is prepared by in-situ micro wire electrical discharge machining (WEDM). This technology focuses on the difficulty of array structure manufacture in micro-electro-mechanical systems (MEMS). A micro-ECM system is built based on the micro-WEDM machine to achieve high precision processing of the array micro-grooves. Since micro-WEDM has good performance in high precision machining of the rotating structure, single and multi-edge disk electrodes can be fabricated in-situ using graphite. The as-prepared disk tool electrode is directly used for micro-electrochemical milling of the array micro-grooves without disassembling away from the device, which avoids the positioning error caused by the re-clamping of the disk electrode. With the advantages of high surface quality and no electrode loss, micro-ECM improves the manufacture performance of the micro-parts. Through wire path optimization, the shape accuracy of the disk edge is improved. After the research of the micro-ECM parameters, the process is improved, and finally, the high precision array micro-grooves are obtained. This method combines the advantages of micro-WEDM and disk electrode micro-ECM milling, and it is convenient for large-scale manufacture of array micro-structures on micro-parts and MEMS.

## 1. Introduction

Micro-electro-mechanical systems (MEMSs) are smaller in size in comparison with traditional mechatronic systems. They are widely used in medicine, military fields, and our daily life [1,2,3]. They have high requirements in parts, not only small size but also complex microstructures [4,5]. Microarrays play an important role in many key components [6,7]. Micro-grooves are popular in bioreactors and microfluidics fields [8,9]. Manufacturing micro-groove arrays with high-precision and large-scale is significant for improving performance of the device and reducing production costs.

Micro-manufacturing technology plays a key role in MEMS. Typical methods include ultra-precision cutting, LIGA technology, laser processing, micro-electrical discharge machining (mic-EDM), micro-ECM and so on [10,11]. These methods can be used in specific case for their advantages, but limitations such as complicated process and expensive equipment restrict their application [12]. Micro-EDM is suitable for the processing of hard and brittle materials and 3D complex curved parts, because there is no obvious cutting force [13,14,15]. However, tool electrode wear in EDM affects precision and efficiency [16]. Micro-WEDM does not require complex shaped electrodes and loss of electrode wire can be ignored [17]. There is no deformation on the workpiece, so it is popular in making milling cutter and grinding edge of turning tool. Research has been carried out on micro-structure and micro-tool manufacture by micro-WEDM. Chen et al. [18] developed a multi-degree of freedom micro-WEDM machine, and it was used to trim the PCD grinding wheel mounted on the high-speed spindle to obtain a very thin cutting edge. Further, various shapes of workpieces were manufactured on the machine with high accuracy and low surface roughness. Schoth et al. [19] manufactured micro-channels and pyramids on stainless steel, ceramics and carbon fibers by micro-WEDM, then these structures were employed in microsurgery, biotechnology and micro fuel cell. ECM is a processing method that utilizes the anode dissolution on the electrochemical reaction to remove material. During the ECM process, there is a gap maintained between the tool electrode and the workpiece, so there is no macroscopic force [20]. Moreover, there is nearly no wear on the tool electrode [21]. Material removal of the workpiece is performed in the form of ions, there is no recast layer on the machined surface, which guarantees the surface quality of the workpiece [22,23]. Nevertheless, stray corrosion of the electrolyte reduces the machining accuracy of ECM, and it is difficult to make tool cathode with complex structure. These problems restrict the extensive application of ECM technology. Kim et al. [24] made disk-shaped tool electrode by micro-EDM, then the 3D micro-cavity without taper was obtained by ECM milling in H_2_SO_4_ electrolyte. Wang et al. [25] researched the micro tool in-situ fabrication, a tool electrode with a diameter of 2 μm was obtained by ECM, then they made a micro-helical structure of 8μm width on nickel sheet.

Micro-parts play a key role in MEMS devices. Array micro-grooves are used in micro plate heat pipes to improve heat transfer performance, and they are also used in micro fuel cells to increase the specific surface area [26,27]. Popular means required to make tool electrode, then manufacture the grooves. However, existing research in making tool electrodes show many drawbacks. The tool cathodes processed by ion beam etching are generally shallow, and difficult to perform complex three-dimensional. And more, electrochemical etching and EDM reverse processing have poor dimensional accuracy. In addition, for micro-manufacture, the size of tool electrode is generally about tens of micrometers, once the tool electrodes away situ after fabrication, clamping error appears when re-clamping for array structure processing. This error seriously affects the precision, and it is extremely detrimental to the performance of the array micro-grooves. 

Based on the characteristics of the array micro-grooves, and aiming at the defects of the existing methods, this paper proposes a method that disk tool electrode is fabricated by micro-WEDM, then in-situ micro-ECM for micro-grooves. A micro-ECM system is developed on the micro-WEDM machine to expand its function. Micro-WEDM shows high precision in the rotary structure machining, so we use it to make disk electrodes with different edge width. CNC system controls the track, then disk electrodes with complex edge profiles or multi-edges can be made flexibly. ECM system is added on the micro-WEDM machine for in-situ micro-grooves manufacture. The disk electrode does not need to be disassembled from the machine, and it can be directly used for ECM. All of the process is carried out in one position aligning, which avoids the error caused by re-clamping, so the dimensional accuracy can be ensured. Further, there is no tool wear during micro-ECM, so this method is efficient and low cost. Through multi-edges disk electrodes, it is helpful for efficient manufacturing array micro-grooves without re-casting layer, surface quality of the workpiece is improved. We optimize the cutting trajectory to obtain a multi-edges disk electrode of high quality, and adjust the parameters of micro-ECM. Finally, array micro-grooves are obtained. This micro-electrochemical milling method based on in-situ disk electrode fabrication provides a new idea in the manufacture of the large aspect ratio grooves. 

## 2. Experimental Details 

### 2.1. Machine Facilities and Materials

**Figure 1:**
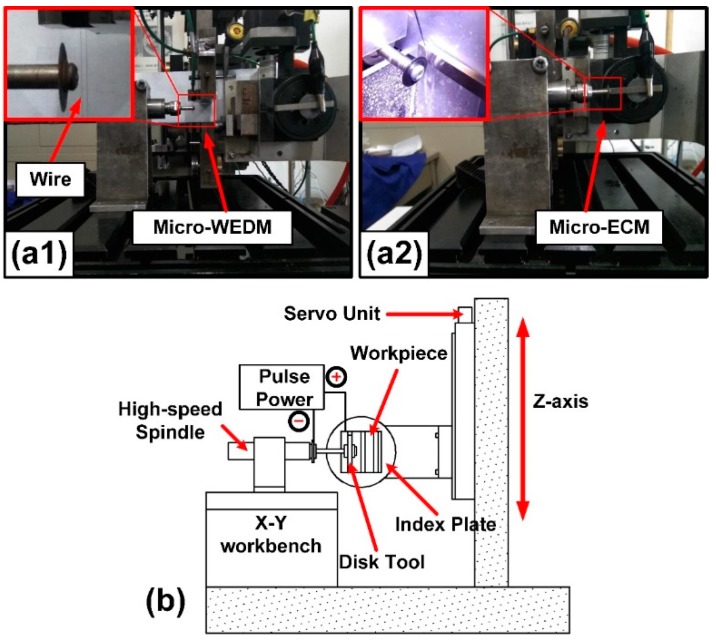
(**a1**,**a2**) Photographs of the micro wire electrical discharge machining (micro-WEDM) machine with high precision micro-electrochemical milling device. (**b**) The device diagram of the micro-electrochemical milling module.

In this experiment, we expand the function of a self-developed micro-WEDM machine. An ECM module is built to realize micro-grooves manufacture, while disk tool electrode is prepared in-situ by micro-WEDM. The devices of disk tool electrode in-situ making and electrochemical milling are in Figure 1. We build a Z-axis based on the X-Y motion platform of the original micro-WEDM machine. On the Z-axis stage, an indexing turntable driven by stepper motor is installed, then the workpiece can rotate at any angle. The movement along the Z-axis is the primary motion of the micro-electrochemical milling. Electrode system is mainly composed of a high-speed electric spindle and a disk electrode. The electric spindle is mounted on the X-Y workbench, and the edge of disk electrode is achieved relying on the motion of X-Y workbench.

A combination of EDM and ECM power source is employed in the whole process. RC micro-energy pulse power source is used for cutting disk electrode. While in micro-ECM, a self-developed high-frequency narrow pulse power source is used. The performance of the ECM power supply is the main factor affecting the machining quality. This ECM power supply can provide high-frequency pulse current. Direct current ECM is easy to cause large-scale stray corrosion, while pulse current can improve the localization of micro-ECM. Therefore, we try to adjust higher frequency current so that the stray corrosion is controlled within a small range, improving the ECM capability.

The electrolyte system is mainly composed of liquid storage tank, micropump, filter and flow meter. It is able to rapidly update the electrolyte in the machining gap to improve the processing precision. In order to get high flow rate and pressure at the outlet of the electrolyte, a needle of medical infusion set is employed in the discharge port. Micro diaphragm pump could provide sufficient pressure, so electrolyte enables to pass through the nozzle with a high flow rate. It reaches a flow of 0.3–0.7 L/min and produces a pressure of up to three bar.

In the experiment, graphite is used to make the disk electrode. The 304 stainless steel is used as the workpiece for the array micro-grooves. During micro-WEDM, kerosene (dielectric work solution, EDM-3, Mobil, Irving, TX, USA) is used as medium. NaNO_3_ and complexing agent EDTA-2Na are the electrolyte dissolved by deionized water.

### 2.2. Experimental Procedure

**Figure 2:**
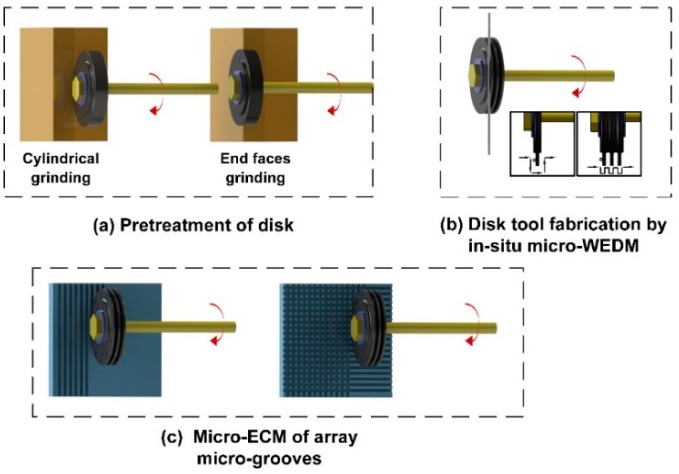
The schematic illustration of the primary fabrication processes that array micro-grooves are obtained by disk tool fabrication and in-situ micro-ECM. (**a**) Pretreatment of disk. (**b**) Disk tool fabrication by in-situ micro-WEDM. (**c**) Micro-ECM of array micro-grooves.

The schematic illustration of this array micro-grooves process is shown in Figure 2. The main procedure includes: (1) pretreatment of disk; (2) disk electrode fabrication by in-situ micro-WEDM; (3) micro-electrochemical milling of array micro-grooves.

#### 2.2.1. Pretreatment and Clamping of the Disk Electrode

Firstly, the graphite is cut into circular sheet with a thickness of 500 μm by high-speed WEDM machine. Then, a 3 mm-diameter through-hole is machined at the center of the circular sheet using EDM drilling, and it is used as mounting hole for the standard rod of the spindle. In order to determine the central position, a location method is proposed: Determining the Y-axis coordinate of the center hole on the two sides of the disk electrode, then aligning the spindle at the y-coordinate line, moving it along X-axis for a distance of one disk radius and one electrode radius, and arriving the center. The graphite disk is mounted on the high-speed electric spindle for block electrode EDM grinding. The thin edge should be grinded from 500 μm to 200 μm. In this step, a large amount of material needs to be removed. Micro-WEDM is low in machining efficiency, so the block electrode EDM grinding before the edge profile machining saves much time, as shown in Figure 2a. The cylindrical grinding is performed first to ensure the concentricity between the excircle and the center hole, then grinding the two end faces, it is sufficient to leave machining allowance of about 10 μm.

#### 2.2.2. Disk Electrode In-Situ Fabrication by Micro-WEDM

**Figure 3:**
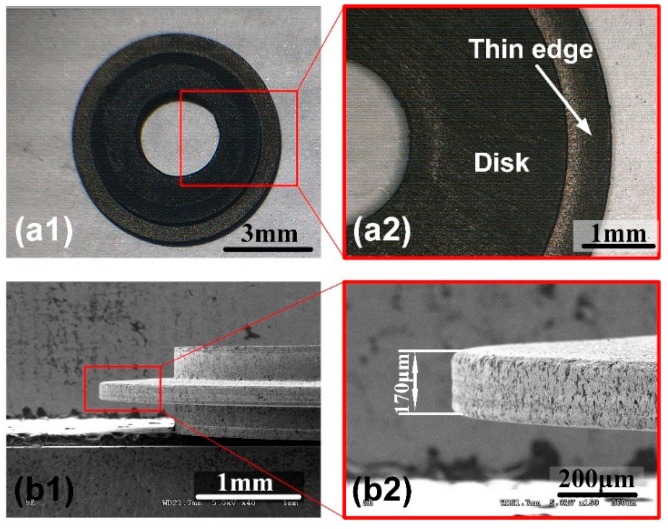
Digital photographs and SEM images of the graphite tool electrode prepared by micro-WEDM with disk high-speed rotation. (**a1**,**a2**) are photograph of the graphite tool electrode and the enlarged view. (**b1**,**b2**) are SEM images of the thin edge of the electrode and the enlarged view.

Due to the uneven wear of the grinding block during the block electrode EDM grinding process, the circular arc would appear at the transition of the square edge, so it is required to remove the reserved 10 μm by micro-WEDM to obtain a better surface quality. Through micro-WEDM technology, the electrode edge is of higher shape accuracy. We can make square and sharp-angled edge disk electrode as well as other profile edge, just using the computer numerical control (CNC) system to design motion trajectory. And more, multi-blade electrode of the desired profile also can be designed. The block electrode EDM grinding and micro-WEDM parameters are shown in Table 1. The discharge energy of the block electrode EDM grinding is larger, and the duty ratio is larger, which can greatly enhance material removal rate and reduce machining time. The digital photograph and SEM image of the graphite tool electrode are shown in Figure 3.

#### 2.2.3. Micro-ECM of Array Micro-Grooves

The study in this paper uses horizontal disk electrode ECM milling, as shown in Figure 2c. After the disk electrode in-situ fabrication, without disassembling disk tool electrode, we can manufacture micro-grooves in Z-axis directly. First, a square single-edge disk electrode is used to research the effect of micro-grooves. The electrolyte is made of 1 wt% NaNO_3_ and 0.5 wt% EDTA-2Na. The Fe^2+^ produced by the anodic dissolution during the ECM would form insoluble precipitate Fe (OH)_2_ with the OH^−^ in the solution, which may clog processing gap or adhesion to the surface of the electrode, affecting the precision and stability. The complexing agent EDTA-2Na makes the precipitate into soluble ions, which greatly improves the stability of ECM. The initial ECM processing parameters are shown in Table 2. Then, we adjust the feed rate, electrolyte concentration and pulse voltage in the experiment, study the influence on the accuracy and surface quality of the micro-grooves. Finally, we select suitable parameters to prepare better array micro-grooves.

### 2.3. Sample Characterizations

The morphologies of the graphite disk electrodes and the micro-grooves were observed by scanning electron microscope (SEM, SU8000, Hitachi, Tokyo, Japan) at 10 kV. The roughness of the surfaces was evaluated by laser confocal microscope (OLS3000, Olympus, Tokyo, Japan). The VHX-600 K (Keyence, Osaka, Japan) three-dimensional optical micro-scope was adopted to observe profiles of the micro-grooves.

## 3. Results and Discussion

### 3.1. Disk Electrode Fabrication by In-Situ Micro-WEDM

**Figure 4:**
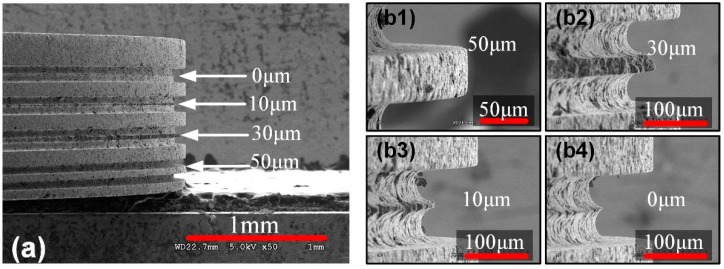
SEM images of the graphite disk electrode by micro-WEDM with the thickness of 10 μm, 30 μm, 50 μm and no offset distance (0 μm). (**a**) SEM image of the as-prepared graphite disk electrode. (**b1**–**b4**) The enlarged view of the thin edges with different width.

The graphite used in this experiment has a grain size of 7 μm, and it can reduce electrolyte corrosion on the disk electrode. When the edge width is about 100–200 μm, shape accuracy is ensured. However, once the edge width is less than 100 μm, large grain size of graphite may affect the quality of thin blade, furthermore, large discharge energy may lead to local deformation on the edge. Therefore, it is necessary to study the thinnest edge width electrode that the graphite could make. SEM images of the graphite disk electrode with different thicknesses are shown in Figure 4. When the width is 50 μm, the edge shows intact profile. Once it is set to 30 μm, 10 μm, or no offset distance (thickness 0 μm), because the grain size of the graphite is 7 μm, discharge energy causes grains fall off thin blade, then a large number of pits appear. There is also vibration of the wire that intensify chipping. It is indicating that graphite is not suitable for the disk electrode with width less than 50 μm. Graphite is employed in square edge electrode with thickness of 50–200 μm.

**Figure 5:**
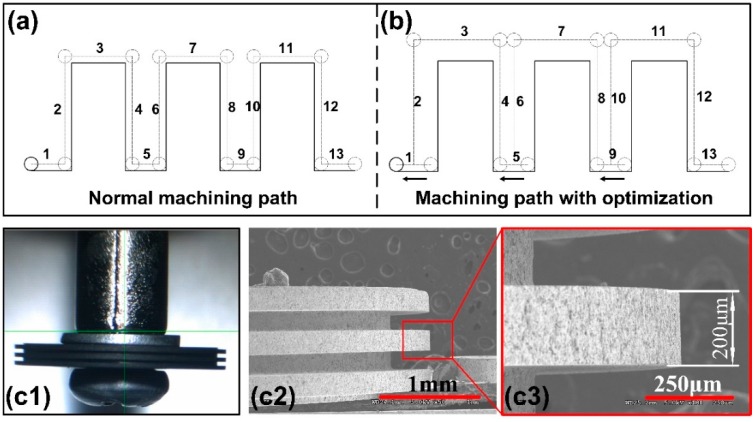
(**a**) The normal machining wire path. (**b**) The machining wire path after optimization. (**c1**) Digital photograph of the 200 μm width multi-edges by optimized path. (**c2**) SEM image of the multi-edges disk electrode. (**c3**) The enlarged view of (**c2**).

Multi-edges electrode could improve the efficiency of micro-electrochemical milling and enrich the shape of micro-structures. During the ECM process, each edge of disk electrode electrochemically reacts with the workpiece, a plurality of micro-grooves would be manufactured. The processing approach of the multi-edge disk electrode is similar to that of the single-edge electrode, so the WEDM parameters can refer to single-edge manufacture. Due to the discharge gap and the diameter of the wire, it is necessary to compensate the CNC path. Generally, the wire path after compensation (without discharge gap) is shown in Figure 5a. There are several problems about this path. The end faces corresponding to the paths 3, 7, and 11 have been smoothed by EDM grinding, the two corners of the path 7 will appear circular arc, and the paths 2,6 and 10 may destroy the machined surface because of the close distance between workpiece and wire. We modify the paths as Figure 5b. The length of the paths 2, 4, 6, 8, 10, 12 is slightly larger than the length of the edge, the paths 2, 6, 10 retract for a certain distance, the paths 3, 7, and 11 no machining, and the 1, 5, 9, and 13 are the main machining trajectories. A three-edges disk tool electrode with an edge width of 200 μm, edge length of 400 μm, and edge pitch of 200 μm is fabricated using the optimized machining path shown in Figure 5(c1–c3).

### 3.2. Experimental Research of Micro-Electrochemical Milling

#### 3.2.1. Influence of Non-Electrical Parameters

**Figure 6:**
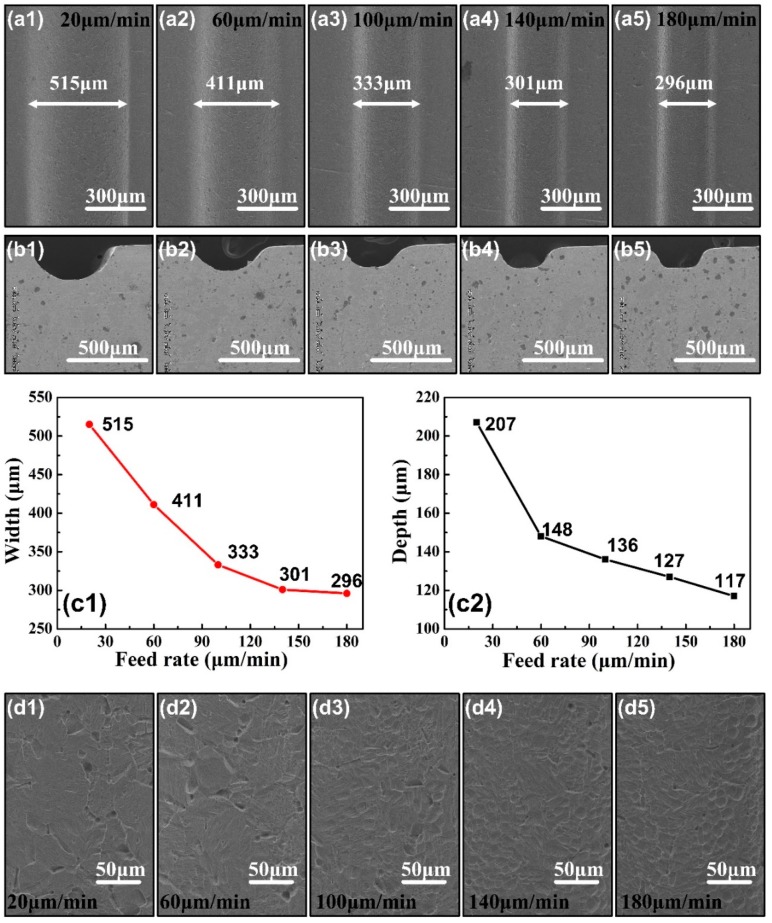
(**a1**–**a5**) SEM images of the morphology on micro-grooves under the feed rates of 20 μm/min, 60 μm/min, 100 μm/min, 140 μm/min and 180 μm/min. (**b1**–**b5**) The cross-profiles of the micro-grooves under corresponding feed rates. (**c1**,**c2**) The width and depth of the micro-grooves changing curves with feed rates. (**d1**–**d5**) The surface quality of the micro-grooves under corresponding feed rates.

In order to study the influence of feed rate on the processing of the micro-groove by disk tool electrode micro-ECM milling, a series of single factor experiments are carried out, and high-speed flushing is used for electrolyte. Changing feed rate, while other parameters are consistent with Table 2. The micro-groove morphologies under different feed rates are shown in Figure 6(a1–a5). The actual profiles of the micro-grooves are in Figure 6(b1–b5). We adopt the upper surfaces of the micro-groove as their width. Since the sidewalls have a certain taper, and the width gradually decreases from shallow to deep, the actual average groove width is less than the size that we measure. The variation of the width and depth of the micro-groove under different feed rates are shown in Figure 6(c1,c2). As the feed rate increases, the width decreases rapidly first, and then the curve becomes flat. In ideal condition, the change of the width is linear with the feed rate. However, in the actual machining, the machining gap cannot maintain ideal state as the feed rate increases. The machining gap reduction leads to the difficulty of the anode erosion products and bubbles expelling in time, which has impact on machining state. When the feed rate is 180 μm/min in the experiment, the occasional retreat duo to short-circuit appears. Frequent retreat of the disk electrode reduces the efficiency of ECM milling. Therefore, corresponding to different processing conditions, there should be an upper limit of the feed rate that exactly doesn’t cause short-circuit retreat. Under this condition, it’s approximated 180 μm/min. When in 180 μm/min, cross-sectional area is about 0.026 mm^2^, and material removal rate is 0.00468 mm^3^/min. While in 20 μm/min, cross-sectional area is 0.082 mm^2^, and material removal rate is 0.00164 mm^3^/min. Therefore, when feed rate is 180 μm/min, the dimensional accuracy is higher, and machining productivity is higher. Figure 6(d1–d5) shows the bottom surface quality of the micro-grooves under different feed rates. When the feed rate is small, the surface quality is slightly improved, but a large number of the stray corrosion pits still distribute on the bottom. We should select the feed rate balanced to the dimensional accuracy and the surface quality in the subsequent research. 180 μm/min is selected in the follow experiments.

**Figure 7:**
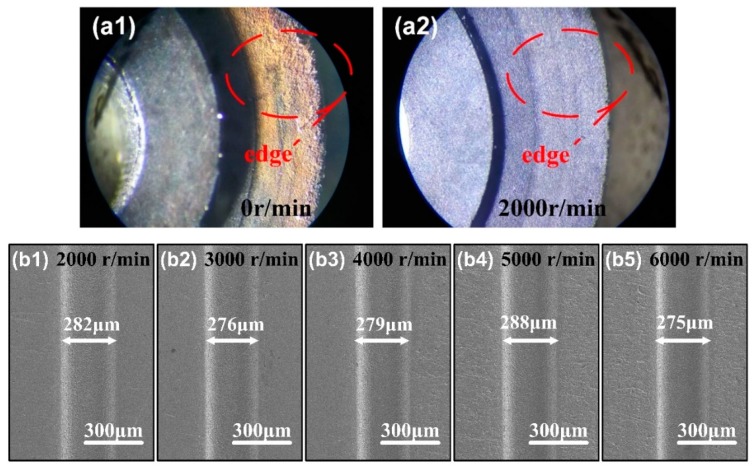
(**a1**,**a2**) Photographs of thin edge surfaces after ECM milling for 30 min with the rotate speed of 0 r/min and 2000 r/min under optical microscope. (**b1**–**b5**) SEM images of micro-grooves under the disk electrode rotate speed of 2000 r/min, 3000 r/min, 4000 r/min,5000 r/min and 6000 r/min.

The minimum speed of the high-speed spindle is 2000 r/min, and we study the influence of the disk electrode rotation. Photographs of thin edge surfaces after ECM milling for 30 min under optical microscope is in Figure 7(a1,a2), the electrode speed is 0 r/min and 2000 r/min, respectively. From the photographs, when no rotation, a layer of rust adheres to surface of the thin edge after some time. EDTA-2Na added in the electrolyte cannot convert insoluble precipitates into ions completely, even under high-speed flushing, a part of undissolved precipitates still adheres to the surface of the disk tool electrode. The rust prejudices reuse of the disk tool electrode, which is bad for surface quality of the workpieces. While the speed is 2000 r/min, there are no precipitates adhering after ECM. Figure 7(b1–b5) are the micro-grooves morphology of micro-electrochemical milling under different electrode speed. With the increase of disk electrode rotate-speed, groove width is hardly changed. The minimum speed of the high-speed spindle is 2000 r/min in this experiment, and the maximum linear velocity has reached 2.1 m/s. Since main function of the high-speed rotation of the electrode is to promote renewal of the machining gap electrolyte, the effect of renewal is sufficient, so the increase of the rotate-speed is not obvious for the improvement of dimensional accuracy and surface quality on the workpiece. On the contrary, the excessive rotate-speed causes larger radial runout error.

**Figure 8:**
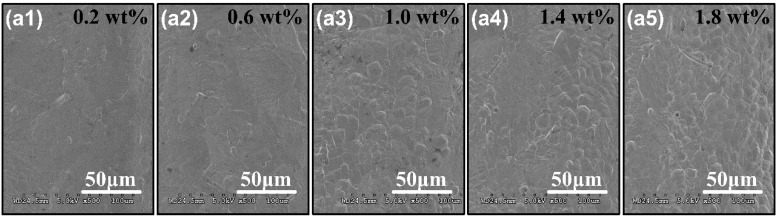
SEM images of the surface quality on the bottom of the micro-grooves with the electrolyte concentrations of (**a1**) 0.2 wt%, (**a2**) 0.6 wt%, (**a3**) 1.0 wt%, (**a4**) 1.4 wt% and (**a5**) 1.8 wt% accordingly.

We select feed rate of 180μm/min and the disk electrode rotate speed of 2000 r/min, then change electrolyte concentrations of NaNO_3_, while others are consistent with Table 2. Figure 8 shows surface quality on the bottom surface of the micro-grooves under different electrolyte concentrations. As the electrolyte concentration decreases, the surface quality gradually becomes better. When the electrolyte concentration is greater than 1wt%, there are a large number of stray corrosion pits on the bottom surface of the micro-grooves. While less than 0.6 wt%, the stray corrosion pits hardly exist, and the surface roughness effectively decreases. We can enhance the surface quality through reducing electrolyte concentration. However, insufficient electrolyte concentration may affect the efficiency of micro-ECM. Therefore, we should use relatively electrolyte concentration on the premise of surface quality. We select 0.2 wt% NaNO_3_ as the best electrolyte concentration in the next research.

#### 3.2.2. Influence of Electrical Parameters

**Figure 9:**
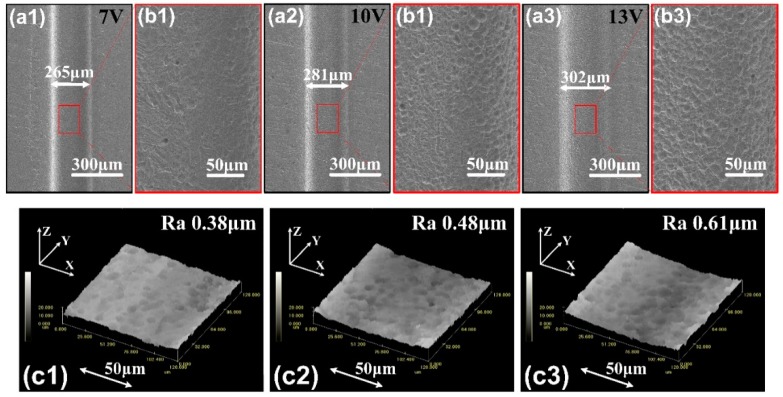
(**a1**–**a3**) SEM images of the micro-groove profiles with the voltage of 7 V, 10 V and 13 V. (**b1**–**b3**) SEM images of the surface quality with corresponding voltage. (**c1**–**c3**) 3D morphologies of the bottom surfaces of the micro-grooves.

When the machining voltage changes, optimal feed rate would change too. In the actual ECM process, the feed rate selects 180 μm/min. When the machining voltage is 10 V, twice short-circuit retreats occur. In contrast, when the machining voltage is 7 V, frequent short-circuit retreats appear, and destroy the machined surface. Under 7 V, we should reduce the feed rate. When the feed rate reduces to 100 μm/min, there is no short-circuit. Figure 9 shows the micro-grooves under different voltages. As the processing voltage decreases, the groove width gradually becomes smaller. We know that the smaller the voltage is, less the machining balance gap. The roughness of bottom surfaces is in Figure 9(c1–c3), when in 13 V, the roughness is Ra 0.61 μm, in 7 V, it is Ra 0.38 μm, much lower than that in 13 V. Aiming at the higher micro-electrochemical machining accuracy, the electrolytic machining voltage should be smaller in the machinable range.

**Figure 10:**
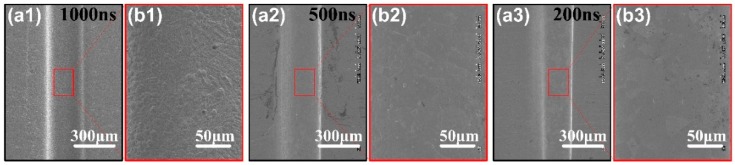
(**a1**–**a3**) SEM images of the micro-groove profiles with the pulse width of 200 ns, 500 ns and 1000 ns. (**b1**–**b3**) SEM images of the surface quality with corresponding pulse width.

The most important factor influence on micro-ECM technology is pulse width. High-frequency narrow pulse is the key to improve the machining precision. We should make sure suitable pulse width corresponding to the groove size, that is the charging time of the electric double layer in a period. The electric double layer charging is completed only in the processing region during pulse width, and a significant electrochemical reaction occurs. Non-processed region doesn’t finish charged, only a weak electrochemical reaction occurs or no react. This transient effect caused by high-frequency narrow pulse technology improves the localization of micro-ECM. As can be seen from Figure 10(a1–a3), when the pulse width is greater than 500 ns, the side gap of the workpiece increases significantly. It is because the charging time of the electric double layer increases, gradually approaching or larger than the time-constant of non-machining region. the transient effect of the narrow pulse ECM is invalid, the groove width increases, and the edge of the micro-groove is no longer sharp. The surface becomes rough, as shown in Figure 10(b1–b3). It is necessary to lower the pulse width within the adjustable range.

#### 3.2.3. Micro-Groove Manufacture by the Optimized Parameters

**Figure 11:**
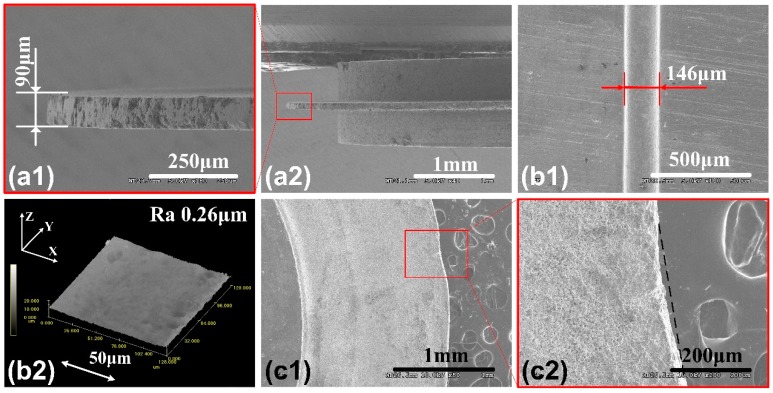
(**a1**,**a2**) SEM images of square-edge graphite disk tool electrode with 90 μm edge width. (**b1**) SEM image of the micro-groove using as-prepared 90 μm width disk electrode under suitable parameters, (**b2**) 3D morphology of the bottom surface. (**c1**,**c2**) SEM images of used electrode.

Through researching the influence of non-electrical and electrical factors, combined with the characteristics of the disk electrode edge profile, the suitable parameters are employed to machine high-quality micro-groove in consideration of the processing efficiency, precision, and surface quality. We use a single-edge disk tool electrode with the edge width of 90μm for micro-ECM, under the conditions of disk electrode rotate-speed in 2000 r/min, pulse voltage in 7 V, pulse width in 200 ns, pulse period in 1000 ns, electrolyte concentration of 0.2 wt% NaNO_3_ + 0.5 wt% EDTA-2Na. Due to the less edge width and pulse voltage, feed rate should reduce to 40 μm/min accordingly. Figure 11 are SEM images of disk electrode and corresponding micro-groove. It can be seen that the micro-groove width is 146 μm, the edge of the micro-groove is clear and sharp. The machining gap is 28 μm. The edge width of the disk electrode reduces, material removal reduces at the same processing depth, so the optimum feed rate increases, the balance machining gap reduces. Roughness of the bottom surface is Ra 0.26 μm, so surface quality is good. It is found that high-frequency pulse is more suitable for micro-ECM. The disk electrode after ECM is in Figure 11(c1,c2), there is some damage in the edge, because long time impact by high-speed flushing electrolyte, some graphite grains fall off. In ECM, cathode does not wear during reaction, the disk electrode can maintain original profile after long time machining.

### 3.3. Array Micro-Grooves Manufacture by Multi-Edges Disk Electrode

**Figure 12:**
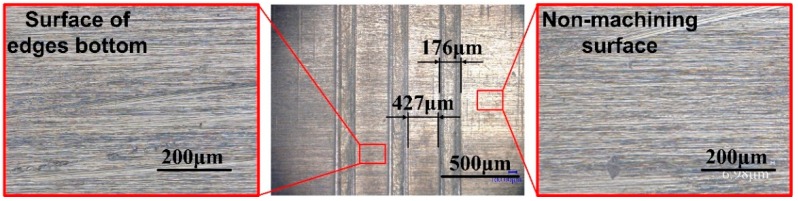
Photographs of the array micro-grooves using three-edges disk tool electrode.

The quality of the multi-edges disk electrode is similar to the single-edge one, which can meet the requirements of micro-ECM milling. We use three-edges, 100 μm edge width, 900 μm edge length and 520 μm interval of disk electrode for experiment. The morphology of the three-grooves is shown in Figure 12. The width is about 176 μm and interval about 427 μm. There is no obvious stray current corrosion at the region corresponding to edges bottom, similar to the non-machining area, so the as-prepared three-grooves is eligible. The multi-edges disk electrode broadens the form of micro-ECM milling, we can manufacture array micro-grooves of different shapes, different depths and different interval at one time. This method verifies the advantages of disk electrode relative to cylindrical electrode on ECM milling.

**Figure 13:**
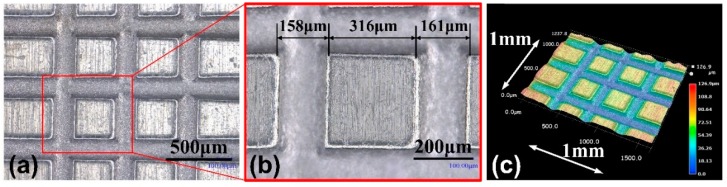
(**a**,**b**) Photographs and (**c**) 3D morphology of the array micro-grooves by two directions micro-electrochemical milling using indexing device.

Figure 13 is the array micro-grooves prepared by multi-edges disk electrode with edge width of 90 μm. The multi-edges disk electrode makes micro-grooves first along X-axis, then turning the workpiece by 90°, continue ECM process. Figure 13b is the local enlargement. The width of the left groove is 158 μm, and the right one is 161 μm. Since the high-speed flushing is by single needle, it is difficult to ensure the same flushing condition on each groove. The array micro-structures may collapse in the corners. Figure 13c is the 3D morphology, the depths of the groove are similar. When the edge width is 90 μm, the dimensional uniformity deviation is 1.89%, and the shape accuracy of the square is high. Multi-needles could adopt in the device for better flushing to improve machining quality. The as-prepared array micro-grooves indicate that the method of micro-electrochemical milling based on disk tool electrode in-situ micro-WEDM making have an excellent application prospect.

## 4. Conclusions

In this paper, a micro-electrochemical milling method based on multi-edges disk tool electrode in-situ micro-WEDM is proposed for array micro-grooves manufacture. Details are as followed:

(1) In this method, the advantages of the micro-WEDM technology in the thin edge rotary structure are utilized perfectly. We can make disk tool electrodes with different thickness. The minimum edge width can reach 50 μm. Through modifying the wire motion path, the time that wire stays on the corner of the edge decreases, through improving path, the edge profiles reach higher accuracy.
(2) A micro-ECM milling module is added on the original micro-WEDM machine, so micro-ECM is carried out directly using as-prepared disk tool electrode without disassembling. It avoids the error caused by re-clamping, improving the processing precision. The influence of parameters and processing conditions on micro-ECM milling are analyzed. Appropriate parameters are selected, then the groove with a width of 146 μm and surface roughness of Ra 0.26 μm is obtained by edge width of 90 μm disk electrode.
(3) Discharge energy is adjusted to make multi-edges disk electrode, then we get the high surface quality array micro-grooves after in-situ micro-ECM milling. The method is high in efficiency and precision, and provides a wonderful technology for the key parts of the MEMS manufacture.

## Figures and Tables

**Table 1:** Block electrode EDM grinding/micro-WEDM parameters.

Parameters	Block Electrode EDM Grinding	Micro-WEDM
Open circuit voltage (V)	200	100
Pulse width (μs)	10	4
Inter-pulse (μs)	10	20
Capacitor (pf)	3300	1500
Servo reference (V)	160	80
Workpiece speed (r/min)	2000	2000
Wire tension (N)	-	1.6

**Table 2:** Micro-electrochemical milling parameters of the single-edge disk electrode.

Parameters	Value
Disk electrode speed (r·min^−1^)	2000
Pulse voltage (V)	16
Pulse width (ns)	2000
Pulse interval (ns)	4000
Electrolyte	1 wt% NaNO_3_ + 0.5 wt% EDTA-2Na
Initial machining gap (μm)	100
Milling depth (μm)	50
Workpiece material	304 stainless steel
Electrode blade width (μm)	170
Feed rate (μm·min^−1^)	100

## References

[1] Cho I., Kang K., Yang D., Yun J., Park I. (2017). Localized liquid-phase synthesis of porous SnO_2_ nanotubes on MEMS platform for low-power, high performance gas sensors. ACS Appl. Mater. Interfaces.

[2] Madinei H., Haddad Khodaparast H., Friswell M.I., Adhikari S. (2018). Minimising the effects of manufacturing uncertainties in MEMS Energy harvesters. Energy.

[3] Zega V., Comi C., Minotti P., Langfelder G., Falorni L., Corigliano A. (2018). A new MEMS three-axial frequency-modulated (FM) gyroscope: A mechanical perspective. Eur. J. Mech. A/Solids.

[4] Ma Z., Zhao H., Du X., Zhou M., Ma X., Liu C., Ren L. (2018). Evaluation of nanoindentation load-depth curve of MEMS bridge structures by calculating the critical elastic-plastic bending deflections. Appl. Surf. Sci..

[5] Yoo K., Park U., Kim J. (2011). Development and characterization of a novel configurable MEMS inertial switch using a microscale liquid-metal droplet in a microstructured channel. Sens. Actuators A Phys..

[6] Lee S., Lee W., Kim H., Bae P.K., Park J., Kim J. (2019). Oscillatory flow-assisted efficient target enrichment with small volumes of sample by using a particle-based microarray device. Biosens. Bioelectron..

[7] Foncy J., Crestel E., Borges J., Estève A., Cau J.C., Vieu C., Malaquin L., Trévisiol E. (2016). Reversible magnetic clamp of a microfluidic interface for the seric detection of food allergies on allergen microarrays. Microelectron. Eng..

[8] Xiang G., Han Y., Wang J., Xiao K., Li J. (2019). A transient hydrodynamic lubrication comparative analysis for misaligned micro-grooved bearing considering axial reciprocating movement of shaft. Tribol. Int..

[9] Chen M., Yang Z., Jin Z. (2019). An experimental investigation of the melting process of an ice bead on the smooth and micro-grooved surfaces under a hot shear flow. Int. J. Heat Mass Transf..

[10] Spieser A., Ivanov A. (2013). Recent developments and research challenges in electrochemical micromachining (µECM). Int. J. Adv. Manuf. Technol..

[11] Rahman M., Asad A.B.M.A., Masaki T., Saleh T., Wong Y.S., Senthil Kumar A. (2010). A multiprocess machine tool for compound micromachining. Int. J. Mach. Tools Manuf..

[12] Ma Y., Liu W., Liu C. (2019). Research on the process of fabricating a multi-layer metal micro-structure based on UV-LIGA overlay technology. Nanotechnol. Precis. Eng..

[13] Chen X., Wang Z., Wang Y., Chi G. (2019). Micro sinking electro-discharge machining of complex 3D micro-cavity using in-situ milled graphite microform electrode. J. Mater. Process. Technol..

[14] Lei J., Wu X., Wang Z., Xu B., Zhu L., Wu W. (2019). Electrical discharge machining of micro grooves using laminated disc electrodes made of Cu and Sn foils. J. Mater. Process. Technol..

[15] Zhao H., Gao C., Wu X., Xu B., Lu Y., Zhu L. (2019). A novel method to fabricate composite coatings via ultrasonic-assisted electro-spark powder deposition. Ceram. Int..

[16] Maradia U., Knaak R., Dal Busco W., Boccadoro M., Wegener K. (2015). A strategy for low electrode wear in meso–micro-EDM. Precis. Eng..

[17] Chen Z., Zhang Y., Zhang G., Li W. (2018). Modeling and reducing workpiece corner error due to wire deflection in WEDM rough corner-cutting. J. Manuf. Process..

[18] Chen S., Lin S. (2011). Development of an extremely thin grinding-tool for grinding microgrooves in optical glass. J. Mater. Process. Technol..

[19] Schoth A., Förster R., Menz W. (2005). Micro wire EDM for high aspect ratio 3D microstructuring of ceramics and metals. Microsyst. Technol..

[20] Nguyen M.D., Rahman M., Wong Y.S. (2013). Modeling of radial gap formed by material dissolution in simultaneous micro-EDM and micro-ECM drilling using deionized water. Int. J. Mach. Tools Manuf..

[21] Yin Q., Wang B., Zhang Y., Ji F., Liu G. (2014). Research of lower tool electrode wear in simultaneous EDM and ECM. J. Mater. Process. Technol..

[22] Luo H., Mi D., Natsu W. (2019). Characteristics of ECM polishing influenced by workpiece corner feature and electrolyte flow. Precis. Eng..

[23] Wu Z., Wu X., Lei J., Xu B., Jiang K., Zhong J., Diao D., Ruan S. (2018). Vibration-assisted micro-ECM combined with polishing to machine 3D microcavities by using an electrolyte with suspended B4C particles. J. Mater. Process. Technol..

[24] Kim B.H., Na C.W., Lee Y.S., Choi D.K., Chu C.N. (2005). Micro Electrochemical Machining of 3D Micro Structure Using Dilute Sulfuric Acid. CIRP Ann. Manuf. Technol..

[25] Wang S., Zhu D., Zeng Y., Liu Y. (2011). Micro wire electrode electrochemical cutting with low frequency and small amplitude tool vibration. Int. J. Adv. Manuf. Technol..

[26] Scotti G., Kanninen P., Matilainen V., Salminen A., Kallio T. (2016). Stainless steel micro fuel cells with enclosed channels by laser additive manufacturing. Energy.

[27] Qi B., Zhou J., Wei J., Li X. (2018). Study on the wettability and condensation heat transfer of sine-shaped micro-grooved surfaces. Exp. Therm. Fluid Sci..

